# Utility of diffusion-weighted imaging to assess hepatocellular carcinoma viability following transarterial chemoembolization

**DOI:** 10.3892/ol.2014.2228

**Published:** 2014-06-05

**Authors:** ZHENG YUAN, WEN-TAO LI, XIAO-DAN YE, WEI-JUN PENG, XIANG-SHENG XIAO

**Affiliations:** 1 Department of Radiology, Shanghai People’s Liberation Army No. 85 Hospital, Shanghai 200052, P.R. China; 2 Department of Radiology, Fudan University, Shanghai Cancer Center, Shanghai 200032, P.R. China; 3 Department of Radiology, Shanghai Chest Hospital Affiliated to Shanghai Jiaotong University, Shanghai 200030, P.R. China; 4 Department of Radiology, Changzheng Hospital, The Second Military Medical University, Shanghai 200003, P.R. China

**Keywords:** hepatocellular carcinoma, chemoembolization, diffusion-weighted imaging, magnetic resonance imaging

## Abstract

The purpose of the present study was to evaluate whether diffusion-weighted imaging (DWI) can be used to assess hepatocellular carcinoma (HCC) viability following transarterial chemoembolization (TACE). A total of 41 consecutive patients were treated according to chemoembolization protocols. The follow-up was performed between six and eight weeks post-chemoembolization by multidetector computed tomography [or enhanced magnetic resonance imaging (MRI)] and DW-MRI on the same day. The presence of any residual tumor and the extent of tumor necrosis were evaluated according to the European Association for the Study of the Liver. The apparent diffusion coefficient (ADC) values of the entire area of the treated mass and the vital and necrotic tumor tissues were recorded. Correlation coefficients were also calculated to compare the percentage of necrosis with ADC values. The mean ADC values of the necrotic and vital tumor tissues were 2.22±0.31×10^−3^ mm^2^/sec and 1.42±0.25×10^−3^ mm^2^/sec, respectively (Mann-Whitney U test, P<0.001). The results from the receiver operating characteristic analysis showed that the threshold ADC value was 1.84×10^−3^ mm^2^/sec with 92.3% sensitivity and 100% specificity for identifying the necrotic tumor tissues. A significant linear regression correlation was identified between the ADC value of the entire area of the treated mass and the extent of tumor necrosis (r=0.58; P<0.001). In conclusion, DWI can be used to assess HCC viability following TACE.

## Introduction

Liver cancer is one of the most frequently diagnosed cancers worldwide, with figures for 2008 estimating 748,300 new liver cancer cases and 695,900 cancer mortalities (1). Furthermore, 50% of these cases and mortalities were estimated to have occurred in China. Hepatocellular carcinoma (HCC) represents the major histological subtype of primary liver cancer, accounting for between 70 and 85% of the worldwide total liver cancer burden (1). The main curative treatment for HCC is considered to be surgical resection, although a number of studies have analyzed the efficacy and safety of a wide array of locoregional therapies (2). Transarterial chemoembolization (TACE) is one such therapy. The technique prolongs patient survival by the arterial injection of anticancer drugs and embolizing agents, resulting in the induction of ischemic necrosis (2). The assessment of the treatment efficacy is vital in determining whether the chemoembolization has been a success and aids in guiding future therapy. Since histological evaluation of each nodule is not feasible or reasonable for patients, the daily practice of monitoring treatment is restricted to the use of radiological imaging to evaluate tumor viability and to reach a conclusion (3). Multidetector computed tomography (MDCT) and magnetic resonance imaging (MRI) are widely used for treatment monitoring. In addition, computed tomography (CT) is commonly used as the standard imaging technique to evaluate the therapeutic response in patients with HCC following TACE. The pattern and distribution of iodized oil in the tumor observed on CT are useful for assessing the effects of TACE. However, the widely used embolizing agent, lipiodol, has the ability to generate considerable artifacts on CT and may therefore change the diagnostic result (4). MRI is more efficient than MDCT in the detection of viable residual tumor elements following lipiodol-based TACE. In addition, with regard to the decision making process following lipiodol-based TACE protocols, the use of MRI is compulsory during the follow-up (4).

Diffusion-weighted imaging (DWI) provides unique information associated with tumor cellularity and cell membrane integrity. Therefore, DWI may be sensitive to the changes that occur in the tumor microenvironment following treatment (5), which can be evaluated quantitatively for the calculation of the apparent diffusion coefficient (ADC). In particular, the degree of tumor necrosis of large HCC following TACE may be predicted by DWI, and patient management may be guided by the results (6). The ADC value may also be used to predict the survival of patients with HCC following TACE (7).

The purpose of the current study was to investigate the ability of DWI to evaluate treatment results with respect to the extent of tumor necrosis and viable tumor tissue following TACE, with a special focus on the feasibility of DWI for the short-term follow-up of HCC following chemoembolization.

## Materials and methods

### Patient characteristics

This study was approved by the Shanghai Cancer Center institutional review board (Shanghai, China) and written informed consent was obtained from all patients. A total of 41 consecutive patients (34 males and seven females; age range, 23–78 years; median age, 56.2±12.5 years) with biopsy-proven HCC (mean diameter, 6.1±2.4 cm; diameter range, 2.5–14.5 cm) were included in this prospective study and treated according to TACE protocols. The demographic, clinical and procedural data is shown in Table I. The follow-up examinations were performed between six and eight weeks post-chemoembolization by MDCT (or enhanced MRI) and diffusion-weighted (DW)-MRI on the same day. Enhanced MRI was performed if the CT showed uncertainties concerning the residual or recurrent metastatic lesions.

### Chemoembolization

Selective chemoembolization was performed with a microcatheter positioned at the hepatic artery branches supplying the tumors. The chemoembolic mixture contained cisplatin (150–300 mg Platinol; Bristol-Myers Squibb, Princeton, NJ, USA), epirubicin (40–50 mg Pharmorubicin; Pharmacia & Upjohn, Milan, Italy), mitomycin (6–10 mg mitomycin-C; Kyowa Hakko Kirin Co., Ltd., Tokyo, Japan) and non-ionic contrast material (6–30 ml Ultravist; Schering, Berlin, Germany) in iodized oil (Lipiodol Ultra Fluide; Laboratoires Guerbet, Aulnay-sous-Bois, France). The dose administered was dependent on the tumor size, hepatic function and health status of the patient. Gelatin sponge particles saturated with contrast medium [5–10 ml of 300 mg/ml iohexol (Omnipaque 300); GE Healthcare, Shanghai, China] were injected into the tumor to slow down blood flow to the tumor following the lipiodol injection. The amount of particulate embolization also varied with underlying factors, such as the presence of the main branch portal or hepatic venous tumor invasion.

### DW-MRI technique

All DW-MRI was performed on a 1.5-T system (GE Signa HD scanner at 1.5 T; GE Healthcare, Amersham, UK) with an eight-channel phased-array body coil. Axial DWI of the liver was performed using the breath-hold single-shot spin-echo echo-planar technique. In total, 18 sections were acquired during each 24 sec breath-hold on inhalation. The image parameters for the DWI images were as follows: Repetition time (TR)/echo time (TE), 1,500/51.6 msec; section thickness, 7 mm; slice gap, 1 mm; number of acquisitions, 2; matrix size, 128×128; field of view, 38 cm; and receiver bandwidth, 166.67 kHz. The diffusion weighting was applied in all directions, with b=0 and 500 sec/mm^2^. The ADC values were calculated by commercially available software and an imaging workstation (both AW4.2; GE Healthcare).

### Enhanced CT and MRI examination

CT evaluation was based on a triphasic (native, arterial and portal venous phases) contrast-enhanced protocol using a 64-row MDCT scanner (LightSpeed VCT, GE Healthcare) at 120 kV, 200 mAs, 0.625-mm collimation and 0.625 pitch in all 41 patients. Arterial-phase scanning was initiated by a bolus trigger technique using a 150 Hounsfield unit threshold, with the region of interest (ROI) placed in the supraceliac abdominal aorta and an additional start delay of 10 sec. The portal venous phase was initiated with a total delay of 50 sec subsequent to achieving the trigger threshold. Non-ionic contrast material (Ultravist 350; Schering) at a dose 80–100 ml was injected via a power injector at a rate 3 ml/sec. Axial images were reconstructed with a 7-mm slice thickness and at 0.5-mm intervals.

Enhanced MRI was performed on 10 patients due to the CT showing uncertainties concerning the residual or recurrent metastatic lesions. MRI was performed with a 1.5T scanner (GE Healthcare), using a sequential acquisition of a 7-mm section thickness. Pulse sequences included a non-enhanced breath-hold T1-weighted gradient echo sequence (TR/TE, 120/1.5 msec; an 80° flip angle; a 320×224 matrix; and a 1–2-mm intersection gap) and a respiratory-triggered fat-saturated T2-weighted fast spin-echo sequence (TR/TE, 4,000–6,000/102–108 msec; four acquired signals; a 384×224 matrix; and a 1–2 mm intersection gap). Contrast-enhanced T1-weighted breath-hold gradient-echo images were acquired in the transverse plane with and without fat saturation using the same technical parameters described for the non-enhanced sequence. For MRI, an intravenous contrast agent (gadodiamide; Omniscan; Nycomed Amersham, Princeton, NJ, USA) was used during dynamic post-contrast imaging.

### Imaging and statistical analysis

The follow-up was performed between six and eight weeks post-chemoembolization. In the follow-up study, responsive lesions were defined as complete and partial response tumors (>50% decrease in the product of the longest diameter and length of the perpendicular diameter of the lesion, or >50% increased necrosis), while non-responsive lesions were defined as stable and progressive disease. Residual viable tumor tissue was considered to be present on enhanced CT or MRI assessment if uptake of the contrast agent was observed in the arterial phase of imaging. In addition, the tumor necrosis response criteria were evaluated based on the modified European Association for the Study of the Liver (EASL) conference (8) by enhanced CT or MRI, while the percentage of tumor necrosis was calculated separately. Lipiodol deposits were defined as constant high-attenuation artifacts in MDCT, without any density changes during the three imaging phases (native, arterial and portal venous) and were rated as tumor necrosis. Qualitative visual assessment was performed blindly by two independent observers (with nine and 30 years of experience, respectively). In the case of a discrepancy in the assessment, the images were reviewed together by the reporting radiologists and a consensus decision was reached.

ADC maps were generated from the DWI, and values were recorded by placing an ROI over the entire area of the treated mass, as observed on the axial image with the maximum lesion size. The ADC values in the viable and necrotic tumor tissue regions of the treated mass were measured by drawing an ROI (≥50 pixels). Correlation coefficients were also calculated to compare the percentage of necrosis on contrast-enhanced CT or MRI and the ADC values. The ADC values of the viable and necrotic tumor tissue regions of the treated mass on the axial image with the maximum lesion size were also recorded.

All statistical analyses were performed with SPSS software (version 10.0; SPSS, Inc., Chicago, IL, USA). Due to unequal variance between the two groups, the ADC values were compared between the viable and necrotic tumor tissues using the Mann-Whitney U test. Receiver operating characteristic analysis was performed to determine a threshold to differentiate the necrotic tumor tissues from the viable tumor tissues. Two-sided tests were used and P<0.05 was considered to indicate a statistically significant difference.

## Results

### Follow-up

Of the 52 target lesions evaluated quantitatively, follow-up examinations were performed between six and eight weeks post-chemoembolization; 36 treated masses were responsive to chemoembolization, whereas 16 lesions were not.

### Qualitative visual assessment following treatment on DWI

**Figure 1:**
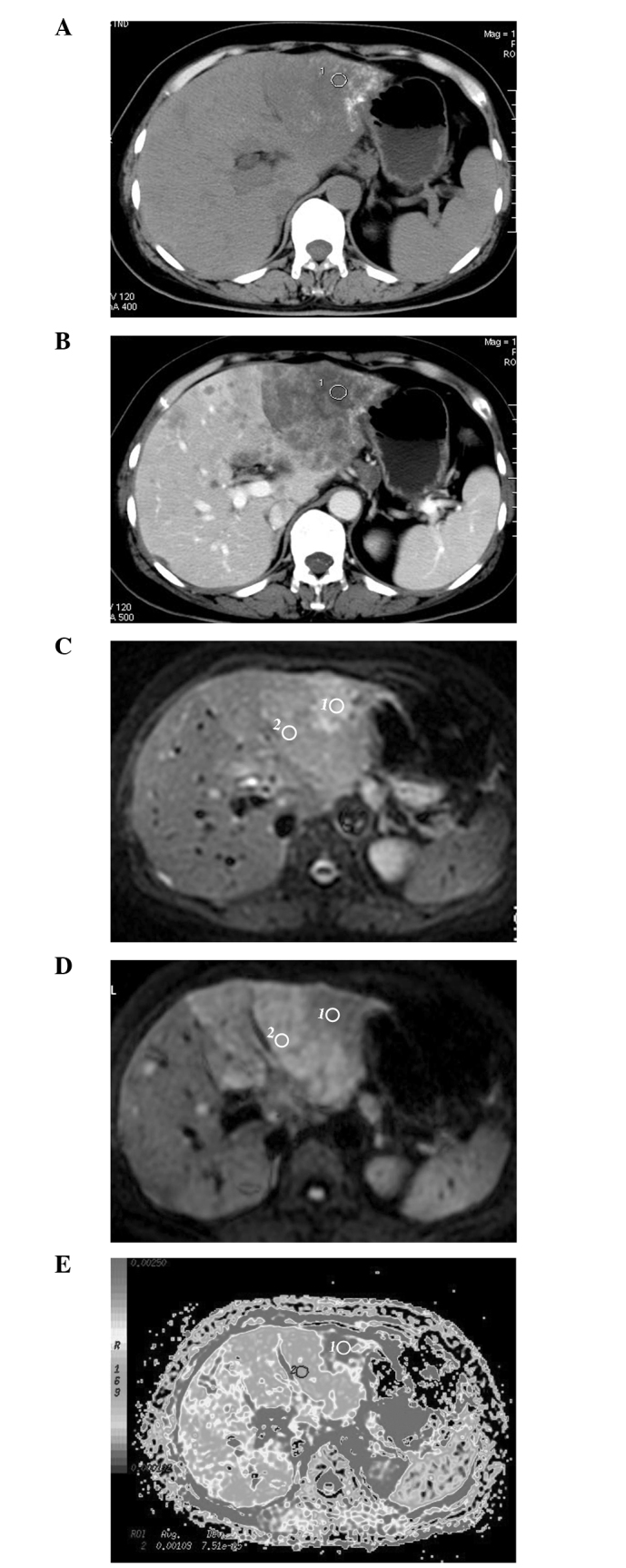
Follow-up CT images eight weeks after lipiodol-based chemoembolization for a 52-year-old female with hepatocellular carcinoma in the left hepatic lobe. CT scan revealed faint accumulation of iodized oil (type III) in the (A) lesion and (B) residual viable tumor with contrast enhancement. Diffusion-weighted magnetic resonance images obtained using b values of (C) 0 and (D) 500 sec/mm^2^. (E) ADC map calculated from diffusion-weighted images. Necrotic components of the tumor (1O) showed greater signal attenuation and higher ADC values compared with the cellular enhancing viable components of the tumor (2O), which restricted diffusion. CT, computed tomography; ADC, apparent diffusion coefficient.

HCC following chemoembolization exhibits variable signal intensities on DWI. Of the 52 treated lesions, six were observed with homogeneous accumulation of the iodized oil on CT images (type I) (9), without any density changes during the three imaging phases or uptake of contrast agent in the arterial phase of the contrast-enhanced examination, which had a decreased uniform signal intensity on DWI obtained with a b value of 500 sec/mm^2^. The other 46 lesions did not show homogeneous accumulation of the iodized oil. A partial defect of the accumulation of the iodized oil (type II) was observed in 28 lesions; faint accumulation (type III) was observed in 10 lesions; and no or slight accumulation (type IV) was observed in eight lesions. In the treated lesions, reduced or increased enhancement (presumed viable) was observed, which demonstrated restricted diffusion (high signal intensity) on the image obtained with a b value of 500 sec/mm^2^, as well as lower ADC values compared with the liver. By contrast, non-enhancing (presumed necrotic) tumors showed higher signal intensities on images obtained with a b value of 0 sec/mm^2^, and greater signal attenuation on images obtained with a b value of 500 sec/mm^2^, with higher ADC values compared with the enhancing tumor (presumed viable; Table II; Fig. 1).

### ADC quantification for viable and necrotic tumors

**Figure 2:**
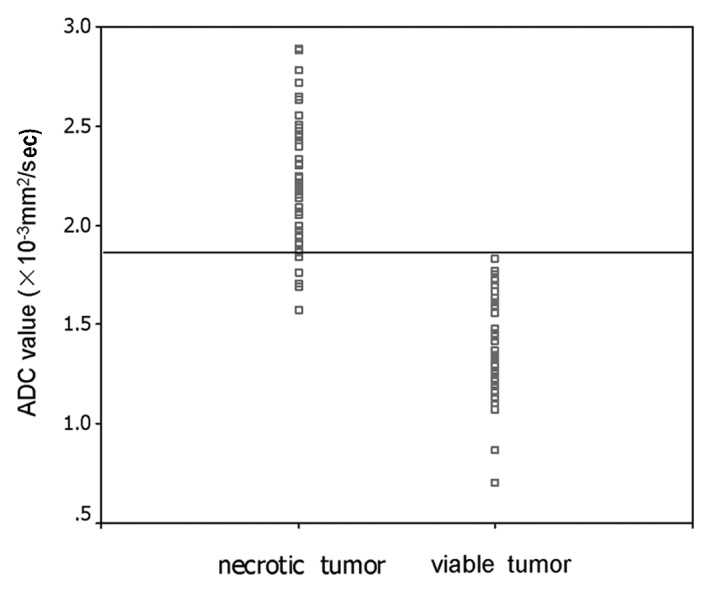
Scatter plots of the ADC values of necrotic and viable tumors. The ADC was significantly lower in the viable tumor than in the necrotic tumor (P<0.001). The line represents the threshold (1.84×10^−3^ mm^2^/sec) for distinguishing the necrotic tumor from the viable tumor using receiver operator characteristic analysis with 92.3% sensitivity and 100% specificity. ADC, apparent diffusion coefficient.

The mean ADC value of non-enhancing (presumed necrotic) tumors was 2.22±0.31×10^−3^ mm^2^/sec (range, 1.57–2.89×10^−3^ mm^2^/sec; median, 2.21×10^−3^ mm^2^/sec), which was greater than that of the enhancing (presumed viable) tumors (mean, 1.42±0.25×10^−3^ mm^2^/sec; range, 0.71–1.83×10^−3^ mm^2^/sec; median, 1.37×10^−3^ mm^2^/sec) (Mann-Whitney U test, P<0.001). The results from the receiver operating characteristic analysis showed that the threshold ADC value was 1.84×10^−3^ mm^2^/sec, with 92.3% sensitivity and 100% specificity for identifying necrotic tumor tissues (area under the curve, 0.985; P<0.001) (Fig. 2).

### Correlation between tumor enhancement and ADC values

The mean ADC value of the entire area of the treated mass on the axial image with the maximum lesion size was 1.92±0.29×10^−3^ mm^2^/sec (range, 1.01–2.57×10^−3^ mm^2^/sec; median, 1.93×10^−3^ mm^2^/sec), while the mean extent of tumor necrosis on contrast-enhanced CT or MRI was 62.6±0.181% (range, 30–95%; median, 65%). A linear regression correlation was identified between the ADC value of the entire area of the treated mass and the extent of tumor necrosis (r=0.58; P<0.001).

## Discussion

In the current study, a significant difference was identified between the mean ADC values of the necrotic and vital tumor tissues (Mann-Whitney U test, P<0.001). In addition, a significant linear regression correlation was identified between the ADC value of the entire area of the treated mass and the extent of tumor necrosis on contrast-enhanced CT or MRI (r=0.58; P<0.001). These results are consistent with previous studies (6,10).

The assessment of the efficacy of HCC following chemoembolization is essential for making therapeutic decisions, such as whether to repeat, interrupt or completely terminate chemoembolization. DW-MRI has the unique ability of being able to provide information that reflects tissue cellularity and cellular membrane integrity (11); this makes it an attractive and useful technique, particularly in those patients with severe renal dysfunction who are at risk from nephrogenic systemic fibrosis (12). Previous studies analyzing the DW-MRI of patients with breast cancer and colorectal patients with hepatic metastases or HCC have shown quantitative DW-MRI findings predictive of the response to chemotherapy (13,14). A study by Kamel *et al* (15) showed that maximum changes in tumor enhancement and ADC values occur between one and two weeks post-therapy. However, the tumor size remained unchanged for up to four weeks post-therapy. In the present study, the preprocedural DWI findings and ADC values were not analyzed. The focus was on the ability of DWI to evaluate responsive and non-responsive tumors during the short-term follow-up. The follow-up was performed between six and eight weeks post-treatment to evaluate the response to treatment by the product of the longest diameter and the length of the perpendicular diameter of the lesion, or according to the modified EASL conference (8) by enhanced CT or MRI.

On DW-MRI, the observation of differential signal attenuation between tissues is useful for the detection and characterization of disease; restricted diffusion (high signal intensity) on higher b value (≥500 sec/mm^2^) images and lower ADC values are demonstrated by cellular tissues, such as tumors or abscesses, whereas greater degrees of signal attenuation on higher b value diffusion images and the return of higher ADC values are shown by cystic or necrotic tissues (16). The visual assessment of DW-MRI, which includes images at higher b values (≥500 sec/mm^2^), may aid to distinguish the different components of HCC (viable and necrotic components) following chemoembolization. As a general observation, necrotic HCC tissues (liquefaction or coagulation necrosis) secondary to chemoembolization typically show a lower signal intensity on higher b value (500 sec/mm^2^) images than viable tissues. However, on diffusion images, the signal intensities observed are dependent on the water proton diffusion and the T2-relaxation time of the tissue, which are possible confounding factors (17). In the current study, on visual inspection of the diffusion images alone, the false-positive identification of necrotic tissue may result from well-differentiated HCC. Therefore, viable and necrotic tumor tissues may occasionally be difficult to characterize with the visual assessment of the DW-MRI alone. As a result, diffusion images must be interpreted concurrently with the ADC measurements to prevent misinterpretation. The diagnostic performance of ADC quantification for the viable and necrotic components of HCC following chemoembolization are also reported in this study. Following chemoembolization, the ADC values of the necrotic tumor tissue were greater than those of the viable tumor tissue (median, 2.21×10^−3^ mm^2^/sec vs. 1.37×10^−3^ mm^2^/sec; Mann-Whitney U test, P<0.001). In viable tumor tissues that are highly cellular, the tortuosity of the extracellular space and the higher density of hydrophobic cellular membranes restrict the apparent diffusion of water protons (18). By contrast, in necrotic tumor tissues, the apparent diffusion of water protons is increased due to cell membrane disruption. Using receiver operating characteristic curves, the current study determined that a mean ADC value of 1.84×10^−3^ mm^2^/sec as the threshold had 92.3% sensitivity and 100% specificity to distinguish the necrotic regions from the viable regions in patients with HCC following chemoembolization.

In this study, with enhanced CT or MRI confirmation of the degree of HCC tumor necrosis following chemoembolization, the ADC value of the treated mass was found to quantify tumor necrosis secondary to treatment. A significant linear regression correlation was also identified between the ADC value of the treated mass and the percentage of tumor necrosis (r=0.58; P<0.001). Kamel *et al* (6) reported that DWI quantifies tumor necrosis following chemoembolization to a greater degree than gadolinium-enhanced MRI. In addition, the ADC values exhibited a higher correlation with the degree of tumor necrosis at pathology (r=0.95; P<0.05) than on gadolinium-enhanced MRI (r=0.55; P=0.12). However, Manelli *et al* (10) showed that compared with DWI, contrast-enhanced MRI with subtraction technique exhibited a more significant correlation with the histopathological findings in the evaluation of HCC necrosis following TACE. In addition, Goshima *et al* (17) stated that DWI was not a reliable predictor of local HCC recurrence following TACE when compared with gadolinium-enhanced MRI. The present study did not evaluate the correlation between ADC values and the degree of tumor necrosis at pathology due to the lack of histopathological examination, which is a limitation of the study.

A number of imaging sequences exist to examine the liver, however, the breath-hold single-shot spin-echo echo-planar technique was used in the present study. The imaging of the liver using this technique is rapid and, dependent on liver size and sequence parameters, it allows the evaluation of the whole liver usually within one or two breath holds of 20–30 sec each. However, a poorer signal-to-noise ratio (SNR), greater sensitivity to distortion and ghosting artifacts, and lower spatial resolution are disadvantages of the breath-hold imaging technique, along with the limitation on the number of b value measurements. Respiratory-triggered DW-MRI has also been shown to improve liver detection in comparison with the breath-hold DW-MRI technique (93.7 vs. 84.3% sensitivity, respectively) (19), with improved image quality, SNR and ADC quantification (20). Spin dephasing occurs as a result of cardiac motion in the left lobe of the liver, which results in the generation of artifacts, particularly at high b values. Furthermore, the use of breath-hold imaging results in artificially high ADC values over the left hepatic lobe (21). Methods of minimizing the occurrence of such artifacts include the use of pulse (22) or cardiac triggering (23) at image acquisition. However, these advanced DW-MRI acquisition techniques were not used in the present study.

Finally, in the process of the follow-up of HCC patients following chemoembolization, the detection of novel tumor nodules and extrahepatic tumor spread are also essential for making therapeutic decisions. In the present study, the ability of DWI for detecting novel HCC and extrahepatic tumor spread was not evaluated. However, several studies have reported that the use of DW-MRI generally results in improved liver lesion detection (24,25).

In conclusion, DWI can quantify HCC tumor necrosis following chemoembolization, and the ADC value may be useful to determine necrotic and viable tumor tissues. Additionally, DW-MRI shows improved liver lesion detection. Therefore, DWI may be an option for the short-term follow-up of HCC patients following chemoembolization and may guide patient management for reducing radiation exposure of CT examination and the risk of contrast material-induced nephropathy.

## Figures and Tables

**Table I:** Demographic, clinical and procedural data.

Data	Value
Age, years	
Mean ± SD	56.2±12.5
Range	23–78
Gender, n (%)	
Male	34 (83)
Female	7 (17)
Child-Pugh class, n (%)	
A	15 (37)
B	26 (63)
C	0 (0)
Morphology, n (%)	
Unifocal	32 (78)
Multifocal	9 (22)
Cirrhosis, n (%)	
No	5 (12)
Yes	36 (88)
Tumor size, cm	
Mean ± SD	6.1±2.4
Range	2.5–14.5
Tumor margins, n (%)	
Capsulated	38 (73)
Infiltrative	14 (27)

SD, standard deviation.

**Table II:** Characteristics of treated hepatocellular carcinoma on DWI.

Tumor type	Enhanced CT or MRI	DWI (b=500 sec/mm^2^)	ADC map
Viable tumor	Uptake of contrast agent	Higher signal intensity	Lower ADC
Necrotic tumor	No uptake of contrast agent	Lower signal intensity	Higher ADC
Iodized oil accumulation tumor	No uptake of contrast agent	Lower signal intensity	Higher ADC

DWI, diffusion-weighted imaging; CT, computed tomography; MRI, magnetic resonance imaging; ADC, apparent diffusion coefficient.
